# Phylodynamic Inference across Epidemic Scales

**DOI:** 10.1093/molbev/msx077

**Published:** 2017-02-14

**Authors:** Erik M. Volz, Ethan Romero-Severson, Thomas Leitner

**Affiliations:** 1 Department of Infectious Disease Epidemiology, Imperial College London, London, UK; 2 Theoretical Biology and Biophysics, Group T-6, Los Alamos National Laboratory, Los Alamos

**Keywords:** phylodynamics, coalescent, HIV, Ebola

## Abstract

Within-host genetic diversity and large transmission bottlenecks confound phylodynamic inference of epidemiological dynamics. Conventional phylodynamic approaches assume that nodes in a time-scaled pathogen phylogeny correspond closely to the time of transmission between hosts that are ancestral to the sample. However, when hosts harbor diverse pathogen populations, node times can substantially pre-date infection times. Imperfect bottlenecks can cause lineages sampled in different individuals to coalesce in unexpected patterns. To address realistic violations of standard phylodynamic assumptions we developed a new inference approach based on a multi-scale coalescent model, accounting for nonlinear epidemiological dynamics, heterogeneous sampling through time, non-negligible genetic diversity of pathogens within hosts, and imperfect transmission bottlenecks. We apply this method to HIV-1 and Ebola virus (EBOV) outbreak sequence data, illustrating how and when conventional phylodynamic inference may give misleading results. Within-host diversity of HIV-1 causes substantial upwards bias in the number of infected hosts using conventional coalescent models, but estimates using the multi-scale model have greater consistency with reported number of diagnoses through time. In contrast, we find that within-host diversity of EBOV has little influence on estimated numbers of infected hosts or reproduction numbers, and estimates are highly consistent with the reported number of diagnoses through time. The multi-scale coalescent also enables estimation of within-host effective population size using single sequences from a random sample of patients. We find within-host population genetic diversity of HIV-1 p17 to be 2Nμ=0.012 (95% CI 0.0066–0.023), which is lower than estimates based on HIV envelope serial sequencing of individual patients.

## Introduction

Genetic diversity of pathogens is shaped by evolution at multiple scales: within individual hosts, at the level of an epidemic among infected hosts, and within meta-populations of structured host populations. The importance of evolution within hosts was highlighted by Grenfell et al. (2004), who introduced the concept of *phylodynamics* to refer to the study of pathogen evolution arising from the interaction of within-host immunological and between-host epidemiological dynamics. Despite this, with few exceptions (Wakeley and Aliacar 2001; Dearlove and Wilson 2013; Didelot et al. 2014) research in pathogen phylodynamics has neglected the role of within-host evolution. Genetic diversity within hosts is usually assumed to be negligible out of mathematical necessity, since there are very few parsimonious population genetic frameworks that allow for efficient statistical analysis of highly complex multi-scale evolutionary processes. The current deficit in efficient analytical approaches for studying multi-scale phylodynamic processes was recently highlighted as a pressing challenge for the phylodynamics field (Frost et al. 2015).

Assuming negligible within-host genetic diversity has allowed major advances in phylodyanmic methods (Drummond et al. 2005; Minin et al. 2008). Recently developed methods enable the estimation of epidemic reproduction numbers (*R*_0_) (Stadler et al. 2012), transmission rates, and population structure (Rasmussen et al. 2014b). Other approaches have been developed to estimate the unobserved number of infected hosts, which can be done explicitly with coalescent models (Volz et al. 2009; Volz 2012) or implicitly using sampling-birth–death (BD) models by the estimation of sampling rates (Stadler et al. 2012). Phylodynamic inference can also be accomplished by approximate Bayesian computation (Poon 2015).

**Fig. 1:**
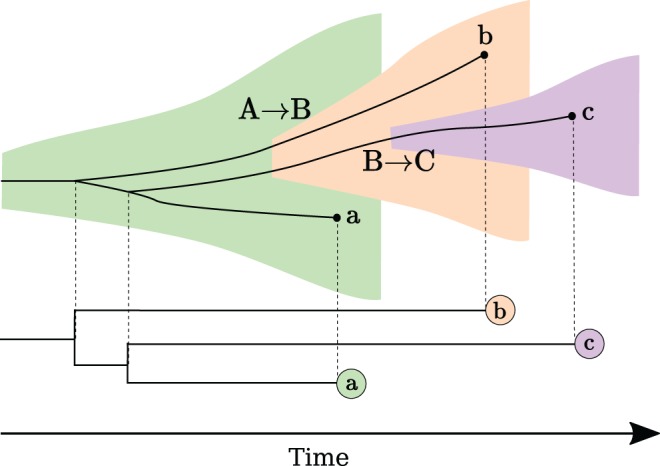
The pretransmission interval and incomplete lineage sorting. The shaded tree represents a transmission chain where each region represents the pathogen population in each of three patients. The width of the shaded regions corresponds to the genetic diversity. In this scenario, A infects B with an imperfect transmission bottleneck, and then B infects C. The genealogy at the bottom is reconstructed from a sample of a single lineage from each patient at three distinct time points. When diversity exists in donor A, a pre-transmission interval will occur at each inferred transmission event (MRCA(A,B) precedes transmission from A to B), and the order of transmission events may become randomized in the virus genealogy. Note that the pre-transmission interval also is a random variable defined by the donor’s diversity at time of each transmission. Terminal branch lengths are also elongated due to these processes.

It is presently unclear how unmodeled within-host evolution will bias popular and widely-used phylodynamic inference methods. For example, the phylodynamics of HIV-1 have been intensively studied (Volz et al. 2013), and evolution of HIV-1 within-hosts has been characterized extensively (Leitner et al. 1996; Leitner and Albert 1999; Vrancken et al. 2014), which has shown that basic assumptions of existing phylodynamic inference approaches are not likely to be met in practice (Romero-Severson et al. 2014). Within-host diversity in a donor at time of transmission causes three problems if the pathogen phylogeny is equated with the true transmission history (fig. 1): (1) Internal nodes of the tree are always shifted to the past because transmitted lineages represent a subset of potentially diverse lineages in the donor. This is known as the pre-transmission interval (Leitner and Albert 1999). How much they are shifted depends on the diversity of the donor’s population, which in turn depends on how long the donor has been infected at the time they transmit to a new recipient. (2) When a donor transmits to more than one recipient, it is possible that the second recipient receives an older lineage, which causes incomplete lineage sorting, such that the order of transmissions becomes disordered compared with the transmission history (Romero-Severson et al. 2014). The probability of disordering depends again on the diversity in the donor, and additionally how much time has passed between the separate transmission events. Finally, (3) when transmission involves more than one lineage from donor to recipient, i.e., an imperfect bottleneck, this too may lead to incomplete lineage sorting, and limited sampling in this situation may give different phylogenetic reconstruction results. Thus, within-host population and evolutionary processes add both bias and noise to the relationship between transmission history and pathogen phylogeny making straightforward epidemiologic interpretations of a phylogeny difficult.

In this investigation, we develop a flexible phylodynamic inference framework for estimation of population size and reproduction numbers through time in the presence of non-negligible within-host diversity. The approach is based on a coalescent model for the genealogy and a semi-parametric model for the birth rate through time, and is similar to widely-used skyline estimation methods. In distinction to existing skyline methods, the present approach does not estimate the effective number of infections through time, but rather the unobserved distribution of lineages occupying individual hosts. For example, if there are ten lineages ancestral to a sample, they may occupy anywhere from one to ten distinct infected hosts, and the new approach is based on estimating this distribution as well as the within-host effective population size. By estimating a distribution, as opposed to a single statistic (effective number of infections), this approach can flexibly accommodate a non-negligible within-host effective population sizes and an imperfect transmission bottleneck.

Within-host effective population size is conventionally estimated using serial sequence sampling of individual infected hosts over an extended period of time (Brown 1997; Rodrigo et al. 1999; Dialdestoro et al. 2016). A significant contribution of the new approach is that it enables estimation of within-host effective population size from single-sequencing of pathogen lineages from multiple distinct hosts in an outbreak. We show computationally that within-host effective size is statistically identifiable from commonly available single-sequencing outbreak data.

### New Approaches

We use a BD demographic process with time-dependent birth and death rates to model the number infected through time. This process is described by the following variables: number infected size *y*(*t*), population birth rate *f*(*t*), per-capita transmission rate β(t)=f(t)/y(t), population death rate ω(t) and per-capita death rate γ(t)=ω(t)/y(t). We restrict our focus to parametric models for *f*(*t*) and will generally assume that γ(t) is constant. For phylodynamic inference, we use a family of flexible spline functions for log(f(t)), further described in the Methods section, which can well approximate a range of non-linear epidemic scenarios such as SIR epidemics with herd immunity or seasonal periodicity (Anderson et al. 1992). We refer to this semi-parametric approach as the *skyspline* model, and likelihoods with the skyspline may make use of traditional coalescent models or the multi-scale coalescent model (MSCoM) described below.

With *f*(*t*) and initial infected population size *y*(0) specified, the approximate population size through time can be modeled deterministically as the solution to the ordinary differential equation:

(1) $$\frac{d}{dt}y(t)=y(t)(\beta (t)-\gamma )$$

The reproduction number can also be computed directly from this model:

R(t)=f(t)/(γy(t))

Parameters of the skyspline model are denoted by the vector *θ* and consist of the initial size *y*(0), constant per-capita death rate *γ*, and the parameters of the spline function log(f(t)).

Evolution within hosts is modeled as a neutral coalescent process with constant size *N*. Super-infection (infection more than once from different sources) is disallowed, while co-infection (transmission of more than one lineage) is possible. Every host is infected once and only once. Going backwards in time, at the time of transmission from a donor to a recipient, all extant lineages in the recipient are transferred to the donor representing a (potentially large) transmission bottleneck, causing a dependence of rates of co-infection on *N*. The model therefore accounts for incomplete lineage sorting and the potentially imperfect correspondence between the topology of the unobserved transmission tree and the pathogen genealogy.

The data used for inference take the form of a bifurcating genealogy G reconstructed from a sample of one lineage per *n* distinct patients at given times $\left(t_{1},\ldots ,t_{n}\right)$ and with time-stamped internal nodes $\left({\tilde{t}}_{1},\ldots ,{\tilde{t}}_{n-1}\right)$. Most phylodynamic inference is concerned with estimation of effective population size through time, $N_{e}(t)$ (Minin et al. 2008). Here, we are focused on connecting effective population size to the true number of infected hosts, and are particularly interested in how phylodynamic estimates of *y*(*t*) are biased by model-misspecification of the epidemiological dynamics and by neglecting within-host evolution. Phylodynamic estimation of *y*(*t*) as opposed to $N_{e}(t)$ can be accomplished using coalescent frameworks such as described in Volz et al. (2009), Frost and Volz (2010), and Volz (2012), sampling-BD models (Stadler et al. 2012), or approximate Bayesian techniques (Poon 2015). We will build on the approach described in Volz (2012). According to the coalescent framework in (Volz 2012),

(2) $$N_{e}(t)=\frac{y^{2}(t)}{2f(t)}.$$

With a skyspline model for *f*(*t*) and *y*(*t*) and the derived quantity $N_{e}(t)$, the probability density of a genealogy given $N_{e}(t)$ can be computed using conventional techniques (Wakeley 2009) which are further described in Methods section. With the likelihoods defined in terms of $N_{e}(t)$, phylodynamic inference can be accomplished using a variety of techniques, including maximum likelihood (see Methods section). We will refer to this model of $N_{e}(t)$ as the CoM12 model (Volz 2012).

The CoM12 model for $N_{e}(t)$ was derived under a number of assumptions, including large population size *y* and assuming nodes in a time-scaled genealogy correspond exactly to the times of transmission events. This latter assumption, discussed in greater detail in Romero-Severson et al. (2014), is valid if within-host diversity of a pathogen is negligible. When it is not, times of common ancestry will precede times of transmission between hosts (fig. 1). The pre-transmission interval together with incomplete lineage sorting may seriously mislead the epidemiological interpretation if host diversity is not accounted for; order and timing of events can be very different in the virus genealogy compared with the actual transmission history. We now derive an approximate coalescent model that accounts for non-negligible within-host diversity (*N* > 0) as well as non-linear epidemic dynamics as specified by the skyspline model. We will refer to this as the MSCoM.

Dynamic variables can be defined on both a forward time axis denoted *t* and a retrospective time s=T−t where *T* is the time of the most recent sample. We then make the following definitions:

- $t=(t_{1},\ldots ,t_{n})$ and $s=(s_{1},\ldots ,s_{n})$ define the times of sampling for each lineage. We assume that each lineage is sampled from a unique host. The sequence $\bar{s}=({\bar{s}}_{1},\ldots ,{\bar{s}}_{n-1})$ are the sorted internal node times in G. And, $\tilde{s}=({\tilde{s}}_{1}\ldots {\tilde{s}}_{2n-1})$ is the sorted sequence of sample and node times in G.
- *A*(*s*) is the number of extant lineages in the genealogy at time *s*
- *B*(*s*) is the number of hosts ancestral to the sample at time *s*; this is the number of infected hosts with at least one lineage that has sampled descendants.

Note that if within-host diversity is negligible, A(s)=B(s), but when it is not, B(s)<A(s). Also note that *A*(*s*) is observed from the tree, which is assumed known. *B*(*s*) is not, and we present one strategy for inferring this. The time argument will be dropped when time-dependency is clear.

At some time *s*, there may be a number *B*_1_ hosts occupied by a single lineage, *B*_2_ hosts occupied by two lineages, and generally *B_k_* hosts occupied by *k* lineages ancestral to the sample with the constraint that $\sum kB_{k}$ equals the total number of extant lineages *A*. Because evolution within hosts is modeled using a neutral coalescent process with constant size *N* in each deme, the coalescent rate among all *A* lineages is

(3) $$\lambda =\frac{B_{2}\left(\begin{array}{c} 2\\ 2 \end{array}\right)}{N}+\frac{B_{3}\left(\begin{array}{c} 3\\ 2 \end{array}\right)}{N}+\cdots =\sum_{k\geq 2}\frac{B_{k}\left(\begin{array}{c} k\\ 2 \end{array}\right)}{N}$$

With the coalescent rate defined in terms of the lineages through time and *s* (Equation 3), the probability of a genealogy is computed in terms of its internode intervals. This is the probability of an ordered sequence of time points generated by a point process with time-dependent rates (Wakeley 2009):

(4) $$p(G|\lambda (\cdot ))=\prod_{i=2}^{2n-1}e^{\int_{{\tilde{s}}_{i-1}}^{{\tilde{s}}_{i}}\lambda (s)ds}(1+(\lambda ({\tilde{s}}_{i})-1)I_{\bar{s}}({\tilde{s}}_{i}))$$

where $I_{x}(\cdot )$ is the indicator function.

With the deterministic model for *y*(*t*), we may consider λ(s) to be a deterministic function of *θ*, and so we may write the likelihood function

(5) l(θ|G)=p(G|λ(·))p(λ(·)|θ)p(θ)=p(G|θ)p(θ)

This equation will be used for maximum likelihood or maximum a posteriori inference for all results presented in the article. If *y* is modeled as a stochastic process, inference is still possible with this coalescent model, but is more complex since some strategy must be employed to integrate over the unobserved y|θ (Rasmussen et al. 2014a).

The distribution of *B_k_* changes over the history of the tree, and if the history of the distribution is known the likelihood of the tree can be computed using Equation 5. To understand how the configuration ${\left(B_{k}\right)}_{k>1}$ evolves through time, it is necessary to derive how the distribution changes at transmission events between two hosts ancestral to the sample, how it changes at sampling events, and how it changes when two lineages coalesce within a host. Here, we sketch the main ideas while detailed derivations are provided in Methods section. When a transmission event occurs between two hosts who are occupied by at least one lineage ancestral to the sample, the two sets of lineages occupy a single deme. The rate that hosts ancestral to the sample transmit to one another is modeled using the same framework as in Volz (2012): Given a transmission event in the population which occurs at rate *f*(*s*), the probability that both hosts involved in the event are ancestral to the sample is

$$\frac{B(s)}{y(s)}\frac{B(s)-1}{y(s)-1}\approx B(s)(B(s)-1)/y{\left(s\right)}^{2}$$

where the denominator is simplified because the epidemic size is generally much larger than the number of ancestral hosts. If the donor *u* harbors *k_u_* lineages and the recipient harbors *k_v_* lineages, then ${\left(B_{k}\right)}_{k>1}$ undergoes the following transformation:

$$B_{k_{u}}\to B_{k_{u}}-1$$

$$B_{k_{v}}\to B_{k_{v}}-1$$

$$B_{k_{u}+k_{v}}\to B_{k_{u}+k_{v}}+1$$

If the donor and recipient are selected randomly without replacement from the population of *B* ancestral hosts, then *k_u_* and *k_v_* follow a hypergeometric distribution. Then *k_u_* and *k_v_* will have covariance which is $O(1/B^{2})$. Now we make the approximation that *B* is sufficiently large that the covariance can be assumed negligible. In that case, we can work with the normalized variables $b_{k}=B_{k}/B$, and the probability that the transmission event yields a host with *k* lineages is

(6) $$b_{k}=\sum_{k_{u}<k-1}b_{k_{u}}b_{k-k_{u}}$$

It is laborious to derive dynamics in terms of the convolution of these random variables, and we therefore present an approach for computing these changes using generating functions in the Methods section.

Next, consider how *B_k_* changes following a coalescent event. The probability that the event happened in a host with *k* lineages is proportional to the coalescent rate $B_{k}k(k-1)/N$. Then modifying ${\left(b_{k}\right)}_{k>1}$ requires appropriate re-weighting of each element according to the probability of not coalescing. Full details are provided in the Methods section.

Finally, a condition of this model is that at most one lineage is sampled from each host, so that following a sampling event, a new host with a single lineage is added and $B_{1}\to B_{1}+1$.

## Results

### Simulated Data

**Fig. 2:**
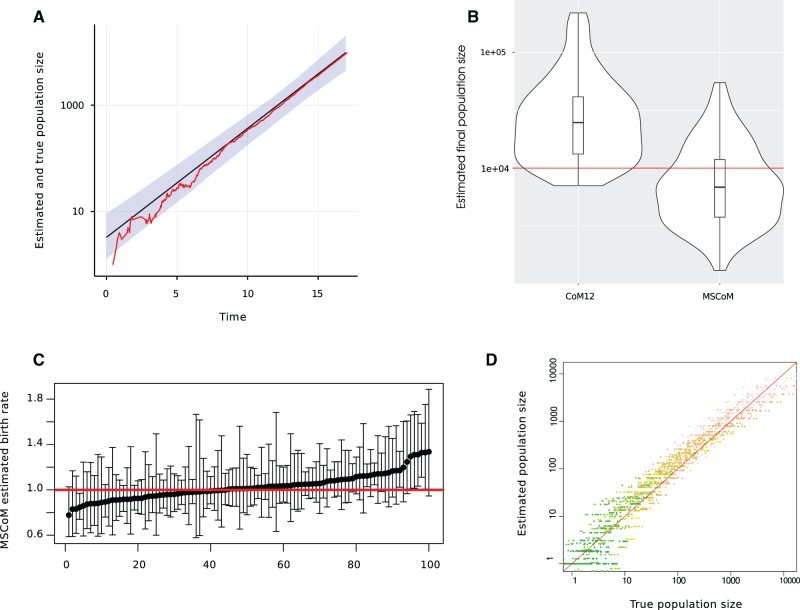
Estimation of population size and transmission rates from simulated pathogen genealogies in a stochastic exponentially growing epidemic with large within-host effective population size. Model parameters are described in the text. (*A*) Example epidemic trajectory (red) and estimated number infected through time (black). Shaded region shows 95% using parametric bootstrap. (*B*) Distribution of the estimated population size at the last sample point using both traditional coalescent model (CoM) and the new MSCoM. (*C*) Estimated transmission rates using the MSCoM across all simulation replicates with 95% CIs based on parametric bootstrap. The red line shows the true transmission rate. (*D*) Comparison of the estimated (MSCoM) and true population size across all simulation replicates. Colors indicate time in the epidemic when the population size comparison is made. Green corresponds to the early epidemic and red corresponds to the late epidemic.

Accurate and precise estimates of transmission rates, population size, and within-host effective population size are obtained with the MSCoM when fitting to genealogies generated by a stochastic BD exponential growth process and when the population size is sufficiently large for the deterministic model to approximate well the true stochastic epidemic trajectory (fig. 2). These estimates are based on simulated data with very large within-host effective population size; in units of coalescent time, the size is equivalent to the duration of four infectious periods on average (N=4/γ). The birth rate was 2γ and sampling proportion was small (<1%) with the final sample being collected when the epidemic had generated 10,000 deaths. In this case, the epidemic trajectory is well approximated by a deterministic exponential function. Computational results suggest that the within-host effective population size is weakly identifiable from the genealogy (supplementary figs. S1–S3, Supplementary Material online) in addition to two of the following three parameters: transmission rate *β*, death rate *γ*, and initial population size *y*(0). Estimates of *N* show upwards bias (mean relative error: MRE = 67%) but good coverage (98% for 95% CI using parametric bootstrap). The standard coalescent model which assumes *N* = 0 also provides accurate estimates of the epidemic population growth rate and transmission rate, however the estimated population size has very large upwards bias. A theoretical explanation for why it is possible for CoM12 to estimate growth rates is provided in the Methods section.

Root mean square error (RMSE) of estimated transmission rates were 11.9 and 15.4% with the MSCoM and CoM12, respectively. Coverage of 95% confidence intervals for transmission rates was 91 and 78% for MSCoM and CoM12, respectively. The MRE of the estimated final number infected (*y*(*T*)) was −0.024 and 2.75 with MSCoM and CoM12, respectively. Whereas MSCoM tends to slightly underestimate population size, CoM overestimates in almost all cases and also has more large outliers. In simulation experiments with *N* = 0 (not shown), both MSCoM and CoM12 provide accurate and precise estimates of transmission rates and population size.

While these simulations have demonstrated good performance under ideal conditions (large population size and simple exponential growth), we also investigated performance under challenging conditions such as sampling when epidemic size is small and subject to large stochastic fluctuations. We simulated the BD process over a range of large sampling proportions (up to 80%) and over a range of within-host effective population sizes including very large values (up to four infectious periods in coalescent time). For each simulated genealogy we fitted the CoM12 and MSCoM models by maximum likelihood and estimated the reproduction number and the final number infected at the time of the last sample. Results are summarized in supplementary figures S4–S7, Supplementary Material online. Results of these experiments show that CoM12 and MSCoM are biased for different parameters in different situations: CoM12 shows robust estimation of *R*_0_ even when population size is small and within-host *N* is small, but is biased upwards when within-host *N* is >0. Bias and precision of CoM12 for estimation of *R*_0_ is not strongly affected by sample proportion. In contrast, MSCoM can provide accurate estimates of *R*_0_ when within-host *N* is large, but is more sensitive to sample proportion. When sample proportion is high (e.g., sampling *n* = 100 when there have been 125 epidemic deaths), MSCoM shows substantial downwards bias for *R*_0_. Future work on stochastic skyspline models may indicate if this bias is attributable to unmodeled stochastic fluctuations of the population size. Regarding estimation of population size, both methods tend to overestimate when sample proportion is high, however the upwards bias is much more extreme for CoM12 in common with findings with small sample proportion.

Whereas simulation experiments with the exponential growth BD process very closely match the assumptions of the MSCoM model, we also sought to investigate the performance of MSCoM in a more realistic epidemiological scenario. We conducted 100 simulations of an HIV epidemic model. In contrast to the BD simulations, this features higher sample density (10%) and non-linear epidemic trajectories (exponential growth followed by decline). Transmission rates are not constant, but vary over the course of infection (high during brief acute infection, low during long chronic infection). And, effective population size within hosts is not constant (low during brief acute infection, high during long chronic infection). We evaluated the potential of MSCoM with the semi-parametric skyspline model to infer population size through time *y*(*t*) and reproduction number through time *R*(*t*). In all cases, we sample homochronously long after epidemic peak. Results are illustrated in supplementary figure S8, Supplementary Material online.

Both the CoM12 and MSCoM effectively capture qualitative features of epidemic trends in *y*(*t*) and *R*(*t*), however both have substantial bias with low precision. The use of the multi-scale model did not in general improve performance in this case, indicating that other forms of unmodeled population structure or population heterogeneity can have equal or greater importance than within-host evolution. The MRE of *R*(*t*) averaged over the entire epidemic trajectory was 0.66 and 0.60 using MSCoM and CoM12, respectively. In simulations with zero genetic diversity within hosts (*N* = 0), the MRE is reduced to 0.59 and 0.49 for MSCoM and CoM12, respectively. While CoM12 outperforms MSCoM by the MRE metric, it also has more large outliers and thus greater RMSE: 1.79 for CoM12 versus 1.66 for MSCoM. Results for estimated number infected *y*(*t*) mirror those for *R*(*t*) with RMSE of 2.37 and 2.10 log units for MSCoM and CoM12, respectively.

In HIV simulations, the within host *N* is initially small for a short period (representing early HIV infection, denoted *N_A_*) followed by a large value in chronic infection (denoted *N*_C_) which lasts many years. Because *N* changes over the infectious period, we cannot compute bias or RMSE, however we can assess how well the estimated constant *N* approximates the true dynamic *N*. The multi-scale coalescent tends to produce estimates that fall between the initial and chronic values, but estimates of *N* also have large outliers and the mean estimate of *N* exceeded the true *N*. Specifically, where *N_A_* = 1 and *N*_C_ = 9, the median and mean estimate of *N* was 7.3 and 14.2, respectively.

### Analysis of Latvian HIV Outbreak

We applied the new coalescent models to 227 HIV-1 *gag* p17 sequences from a Latvian outbreak among injection drug users and heterosexual sex partners between 1990 and 2005 (Balode et al. 2004; Balode et al. 2012; Graw et al. 2012). Three different coalescent models were fitted to time-scaled phylogenies computed using least-squares dating (To et al. 2015): We applied a recently-developed Bayesian non-parametric phylodynamic reconstruction (BNPR) method (Karcher et al. 2016), which provides estimates of the epidemic effective population size through time. Next we fit the semi-parametric skyspline CoM12 model which provides estimates of the number infected and reproduction number through time *R*(*t*). And, we fit the new skyspline MSCoM model which accounts for within-host evolution and additionally provides estimates of the within-host effective population size. The CoM12 and MSCoM models were fitted using maximum a posteriori methods; further details on methodology are in the Methods section.

**Fig. 3:**
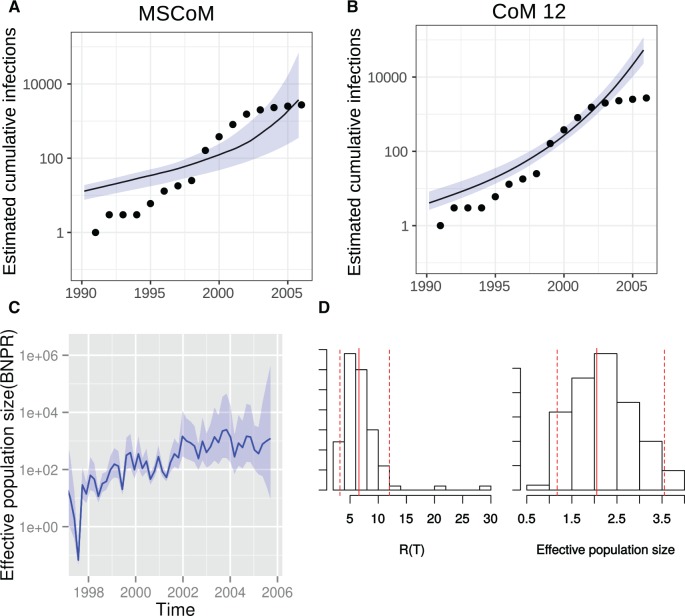
Phylodynamic analysis of 227 HIV-1 *gag* p17 sequences from an outbreak in Latvia showing estimated number infections, effective population size, and *R*_0_. Shaded regions show 95% CIs. (*A*) Estimated cumulative number of infections through time (blue) using the multi-scale coalescent model that accounts for within-host evolution. Points show cumulative reported diagnoses in the outbreak. (*B*) Estimated cumulative number of infections through time (blue) using the coalescent model developed in Volz (2012) that does not account for within-host evolution. (*C*) Estimated epidemic effective population size through time using BNPR (Karcher et al. 2016). (*D*) Estimated posterior reproduction numbers and within-host effective population sizes in units of coalescent time (years). Red dashed lines show 95% interquantile range and solid dash line shows posterior median.

Figure 3 shows estimated cumulative infections, reproduction numbers, and effective population sizes using different methods. Supplementary figure S9, Supplementary Material online, shows estimated number of infections and reproduction numbers through time. A novel aspect of the MSCoM approach is that it provides an estimate of the mean within-host effective population size from a random sample of patients (one sequence sample per host). We can therefore compare these estimates to those obtained by the more common approach of taking numerous serial samples from single hosts. We estimated *N* = 2.05 (95% CI 1.09–3.87) in units of coalescent time (years), which describes the average time to common ancestry for a pair of lineages within a host. The CI width is large, but similar to what was found in simulation experiments with similar sample sizes where it was found that *N* is weakly identifiable. The within-host effective population size of HIV varies substantially over the course of an individual infection, and will also vary substantially between patients. Therefore, this estimate should be treated as descriptive of epidemic-level genetic diversity but not clinically meaningful on an individual basis. More commonly, effective population size is reported in units of population genetic diversity 2Nμ where *μ* is the substitution rate within hosts, and published estimates of within-host 2Nμ for HIV-1 *env* range from 0.04 to 0.144 substitutions/site (Brown 1997; Rodrigo et al. 1999; Seo et al. 2002). The rate of evolution outside of the envelope gene is typically much lower (more than 2-fold) (Berry et al. 2007; Alizon and Fraser 2013), and population genetic diversity will be correspondingly lower in the *gag* p17 gene. We estimate 2Nμ=0.012 (95% CI 0.0066–0.023) using a recent estimate of within-host p17 evolutionary rates by Zanini et al. (2015) of μ=0.003 (range 0.0012–0.0043) substitutions/site/year. Our estimate of the within-host effective population size is lower than previous estimates, which reflects lower evolutionary rates on HIV-1 *gag* then *env*, as well as the fact that these data were sampled from a rapidly expanding IDU outbreak and many patients were not infected for very long prior to sampling. In contrast, previous estimates of 2Nμ are based on HIV-1 *env* sequences sampled from chronically infected patients over many years.

Estimated epidemic growth rates using MSCoM, CoM12, and BNPR estimators were similar, but estimated number infected using CoM12 were substantially larger than MSCoM estimates and generally exceeded the number of diagnosed patients to a large extent. CoM12 estimates were also more unstable and produced more large outliers. Using MSCoM, we estimated a reproduction number in 2005 of *R* = 6.40 (95% CI 3.2–12.0) and using CoM12 we estimated *R* = 13.3 (95% CI 9.1–19.1).

The estimated number infected in 2005 was 2,673 (95% CI 219–50,268) and 42,038 (95% CI 18,200–94,491) with MSCoM and CoM12, respectively. Estimates with CoM12 are not credible since in 2005 there were only 2,728 diagnoses in the IDU and heterosexual risk groups.

### Ebola Virus Outbreak in Sierra Leone

We applied the new coalescent models to Ebola time-scaled phylogenies previously estimated by Gire et al. (2014) in one of the first phylodynamic analyses of Ebola virus (EBOV) during the West African epidemic of 2014. These data were based on 78 whole-genome EBOV sequences collected over approximately one month during the Summer of 2014 in the border regions of Sierra Leone near where the epidemic originated. In contrast to HIV-1, these data represent an outbreak of a pathogen producing acute hemorrhagic fever with a short infectious period and with high transmissibility. The within-host effective population size for EBOV is undocumented to the knowledge of the authors.

There have been two previous phylodynamic modeling efforts of the same data (Stadler et al. 2014; Volz and Pond 2014), which yielded the first estimates of EBOV reproduction numbers for the 2014 epidemic based on molecular data. These analyses neglected, however, potential confounding effects due to unmodeled within-host evolution. Previous analyses of EBOV sequence data have mixed infections with substitutions likely persisting through more than one transmission event, suggesting a large transmission bottleneck (Gire et al. 2014). In this analysis, we evaluate the potential of the new methods to estimate EBOV effective size within hosts and the potential of the skyspline approach to provide a more refined estimate of reproduction numbers through time.

**Fig. 4:**
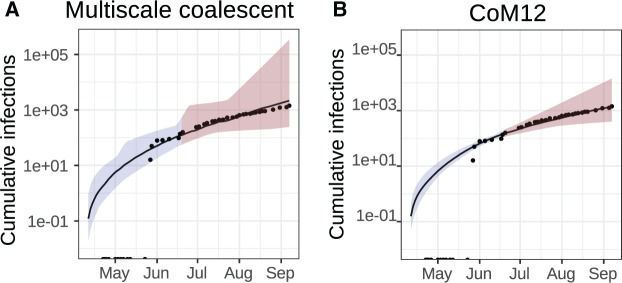
Phylodynamic analysis of time-scaled phylogenies estimated in Gire et al. (2014) based on 78 whole genome sequences from EBOV patients in Sierra Leone in 2014 using MSCoM (*A*) and CoM12 (*B*). Trajectories show estimated cumulative infections through time. The shaded region shows 95% CIs. The red shaded region shows a prediction over a time period where no sequence data were collected. Points show cumulative WHO case reports in Sierra Leone.

Figure 4 illustrates estimated cumulative number of infections through time using the MSCoM and CoM12 models. Supplementary figure S10, Supplementary Material online, shows the number of infections and reproduction numbers through time using both models. Both estimators show concordance with the number of cases reported by the World Health Organization (points), and WHO case reports were not used for model fitting or calibration. Note that up until 18 June, ∼60% of probable EBOV infections were sequenced and that the sequence sampling rate varied dramatically through time (Volz and Pond 2014). The true number of infections is unknown. Sequence data were collected up until 19 June 2014, and the red shaded region shows an extrapolation from the fitted model to a time horizon beyond when sequence data were collected (up to 9 September 2014). Estimates produced by MSCoM and CoM12 are highly similar, with the greatest difference being the size of the estimated credible interval that is due to the estimation of an additional parameter with the MSCoM (*N*). The estimated cumulative number of cases at the time of the last sample in late June is 117 (95% CI 53–412) using MSCoM and 140(95% CI 109–185) using CoM12. The actual number of cases reported by the World Health Organization on June 18, 2014 was 136. The small difference in median estimates is likely due to the relatively small within-host *N* estimated with MSCoM: *N* = 0.16 (95% CI 0.007–3.49) in units of coalescence time (days).

The previous analyses of these data (Stadler et al. 2014; Volz and Pond 2014) were based on models with constant transmission rates and death rates, and as such could not detect changes in the reproduction number over the course of the outbreak. The skyspline approach, however, allows *R*(*t*) to vary smoothly over the outbreak, and we find that the early reproduction number was much larger than at the time of the last sample denoted *T*. We estimate R(T)=1.36 (95% CI 0.82–2.14) with MSCoM and R(T)=1.27 (95% CI 1.03–1.57) with CoM12. The noisiness and non-constancy of *R*(*t*) may partially explain discrepancies between early published estimates of *R*_0_, which were often found to exceed 2, and later estimates of *R*_0_ which were generally <1.75 (King et al. 2015).

## Discussion

The development of a likelihood-based framework for multi-scale coalescent processes opens an interesting avenue for estimating within-host pathogen diversity from data consisting of a single sequence sample from multiple patients in an epidemic. Single sequencing data is far more abundant than serial-sampling data, and serial sampling data is often not available in outbreak situations or with emerging pathogens. We have shown computationally that within host effective population size is identifiable from this type of single sampling data. This may appear surprising in light of population genetic theory developed for stochastic BD processes, which has shown that at most two of three parameters describing a simple BD process will be identifiable from a genealogy: the birth (i.e., transmission) rate, and death rate or population size (equivalently the sampling rate) (Stadler 2009). In addition, our computational results show that the within-host effective population size is weakly identifiable given a genealogy featuring within-host evolution (supplementary fig. S1, Supplementary Material online).

CoM12 and MSCoM make different approximations that can lead to different forms of bias in particular situations. Estimated population size with CoM12 tends to be substantially over-estimated when within-host *N* > 0, yet MSCoM estimates can be biased downwards when the sample proportion is high and when epidemic size is small and subject to large stochastic variation. When estimating *R*_0_, CoM12 is robust to high sample proportion, but not to *N* > 0, and the opposite is the case for MSCoM. When *N* > 0 and sample proportion is high, the probability that more than one lineage will occupy a host is high, making it more important to account for within-host processes with MSCoM. Yet the current implementation of MSCoM is based on a deterministic approximation to the evolution of the number of lineages per host and does not cope well with noisy population dynamics. These results indicate a direction for further extension of the MSCoM approach to stochastic demographic processes, which has already been done for CoM12 (Rasmussen et al. 2014a). In general, when *N* is small, estimates of population size have lower precision using MSCoM, and there is a tradeoff between estimating within-host *N* versus detecting changes in epidemic size. The analysis of EBOV phylogenies shows that estimated population sizes were similar, but MSCoM was less precise. When *N* is extremely large, the ability to infer population dynamics diminishes, since the relationship between transmission events and coalescent events grows weaker.

Analysis of the Latvian HIV-1 outbreak data provides estimates of within-host diversity that are close to estimates obtained from serial sequencing data of individual HIV-1 patients over many years. We conjecture that estimation of within-host effective population size is possible because of the way that the coalescent rate is modulated by the distribution of lineages among hosts. In a standard coalescent process, the coalescent rate changes in a predictable way following a coalescent event: It will decrease by a factor of $\left(\begin{array}{c} A-1\\ 2 \end{array}\right)/\left(\begin{array}{c} A\\ 2 \end{array}\right)=A-2/A$ (Wakeley 2009). In a multi-scale coalescent process, the decrease in coalescent rate depends on the variance in the number of lineages among hosts; if all lineages occupy a single host, the rate will decrease in the same way as the standard coalescent. But if all hosts but one have a single lineage, and one host has two lineages, then the coalescent rate would be zero following the coalescent event, and would not rebound until the epidemic process causes more lineages to be co-located in a single host.

Whether it is of practical importance to consider within-host diversity when conducting phylodynamic inference depends on details of the specific outbreak and pathogen being considered. Analysis of the Latvian HIV-1 outbreak shows that standard coalescent models tend to produce larger estimates of population size than are credible based on independent surveillance data. The multi-scale coalescent process yields estimates that are much closer, but slightly less than the reported cumulative number of diagnoses. In both cases, estimated growth rates in the number of cases are highly consistent with surveillance data, and simulation results suggest that estimates of reproduction numbers will be robust to unmodeled within-host evolution. Good performance of the standard coalescent model for estimating transmission rates in the presence of large within-host effective population size may appear surprising, however this is the prediction of existing theory for coalescent processes in large metapopulations (Wakeley and Aliacar 2001). In the Methods section, we show how the growth rate of the population effective population size is independent of within-host effective size provided transmission rates are constant and there is no super-infection.

In contrast to the HIV-1 outbreak data, analysis of the EBOV outbreak data did not indicate substantial within-host diversity. Estimated population sizes with the multi-scale coalescent were highly consistent with estimates using the standard coalescent and both estimates were very close to the number of cases reported by the World Health Organization over time. The early EBOV epidemic in Western Africa was characterized by several point-source outbreaks originating from unsafe burials (Team 2014). Alternative coalescent approaches such as the lambda-coalescent (Pitman 1999) may also be a useful alternative to the standard coalescent since many lineages will share a common ancestor originating in a single host. The MSCoM implicitly accounts for this as well, since unlike CoM12, times of common ancestry are not presumed to coincide exactly with times of transmission.

While the development of the multi-scale coalescent goes some way towards resolving bias in phylodynamic estimates of the number of infected hosts, other forms of unmodeled heterogeneity can also play an important role. Our simulation results show that even if within-host diversity is negligible, failure to account for variation in transmission rates over an infectious period can substantially bias estimates. Other forms of epidemic-level heterogeneity (different risk groups, geographic structure, age structure, different levels of risk behavior) would presumably also introduce bias into skyspline estimates of epidemic size. Flexible structured coalescent models have been developed which can account for these forms of epidemic-level heterogeneity, however it remains to integrate the structured coalescent model with a parsimonious model of within-host evolutionary dynamics. Future developments on MSCoMs could also incorporate an explicit transmission bottleneck and realistically account for how within-host effective size varies over the infectious period.

## Methods

In this section, we derive the multi-scale coalescent model, describe simulation models, and analysis methods for the HIV-1 and Ebola datasets.

### Multiscale Coalescent Model

*A*(*s*) and *B*(*s*) are the number of lineages and ancestral hosts at time *s* before the most recent sample. $B_{k}(s)$ is the number of hosts harboring *k* ancestral lineages, and $b_{k}(s)=B_{k}(s)/B(s)$ is the proportion of ancestral hosts harboring *k* of *A*(*s*) lineages. *N* denotes the within-host effective size which is constant, and *f*(*s*) and *y*(*s*) are, respectively, the total birth rate and epidemic size through time.

Note that this definition conditions on having at least one lineage, so $b_{0}(s)=0$, and $\sum_{k}b_{k}(s)=1$. We can also define a *probability generating function* (Wilf 2013) for this distribution, and derivations will be easier working with the generating function than using *b_k_* variable for all *k*.

$$g(x;s)=\sum_{k>0}b_{k}(s)x^{k}$$

While generating functions make the derivation more parsimonious, we also provide a derivation for the dynamics of $b_{k}(s)$ without generating functions in the Supplementary Text online.

The mean number of lineages in an ancestral host is $\sum_{k}kb_{k}(s)=g\prime (1;s)$. Note that *B* and *A* are related through the mean number of lineages per ancestral hosts, since we must have g′(1)B=A, and *B*(*s*) is easily defined in terms of *g* and *A*:

B(s)=A(s)/g′(1;s).

This substitution will sometimes be made in the following equations.

Initially, all lineages begin in a distinct host, so $b_{1}(0)=1$ and g(x;0)=x

The coalescent rate can be defined in terms of *g*:

(7) $$\lambda (s)=B(s)\sum_{k}\left(\begin{array}{c} k\\ 2 \end{array}\right)\frac{b_{k}(s)}{N}=B(s)g\prime \prime (1;s)/(2N)=A(s)\frac{g\prime \prime (1;s)}{g\prime \prime (1;s)}\frac{1}{2N},$$

where *N* is the pathogen effective population size within hosts.

Now we can derive the asymptotic dynamics of g(x;s) in the limit of large *A*. The main result is:

(8) $$\frac{\delta g(x;s)}{\delta s}=\frac{A(s)f(s)}{g\prime (1;s)y^{2}(s)}(g^{2}(x;s)-g(x;s))$$

Note that this describes the dynamics of *g* only in internode intervals, and that discrete changes in the distribution will occur at nodes and at sample times in the genealogy. Readers may also refer to the online Supplementary Text online for an alternative derivation of an equivalent system of equations that does not require generating functions.

To derive 8, note that *g* will change when one ancestral host infects another. This occurs at the rate (see Volz 2012).

$$\left(\begin{array}{c} B(s)\\ 2 \end{array}\right)2\frac{f(s)}{y^{2}(s)}.$$

When an ancestral host with *k*_1_ lineages transmits to a host with *k*_2_ lineages it will yield a host with $k_{1}+k_{2}$ lineages. Under the approximation that both *k*_1_ and *k*_2_ are iid from the same distribution generated by *g*, the new host has a number of lineages generated by $g^{2}(x;s)$ (see properties of generating functions in Wilf 2013). In particular, the probability that two randomly chosen hosts will have a total number of lineages equal to *k* is $\sum_{k\prime <k}b_{k\prime }b_{k-k\prime }$ (see Supplementary Material online). In reality, *k*_1_ and *k*_2_ will be correlated, however this correlation will be $O(1/B^{2})$ (following from the hypergeometric distribution and given that we sample *k*_1_ and *k*_2_ without replacement), and if the number of ancestral hosts is large, this will be a good approximation. Concurrently with the transmission event, the hosts with *k*_1_ and *k*_2_ lineages will be replaced with a single host with $k_{1}+k_{2}$ lineages. The total number of hosts will be reduced from *B* to *B* – 1. Thus one out of *B* – 1 hosts will have *k* generated by $g^{2}(x;s)$ and *B* – 2 hosts will have *k* generated by g(x;s). And the size of the change in *g* will be

$$\frac{g^{2}(x;s)}{B(s)-1}+\frac{B(s)-2}{B(s)-1}g(x;s)-g(x;s)$$

Multiplying this change by the rate $\left(\begin{array}{c} B(s)\\ 2 \end{array}\right)2f(s)/y^{2}(s)$ yields Equation 8.

It remains to show how the distribution generated by *g* undergoes discrete changes at nodes in the tree and at sample times.

At an internal node of the tree, a host with *k* lineages is reduced to *k* – 1 lineages and the probability of a particular host with *k* lineages losing a lineages is proportional to k(k−1)/g″(1;s). The probability that any host with *k* lineages loses a lineage is $q_{k}=b_{k}k(k-1)/g\prime \prime (1;s)$. Recall that the number of hosts with *k* lineages is $B_{k}=Bb_{k}$. The following may occur:

- With probability *q_k_*, $B_{k}\to B_{k}-1$
- With probability $q_{k+1},\;B_{k}\to B_{k}+1$
- With probability $1-q_{k}-q_{k+1}$, *B_k_* is unchanged.

Tabulating these events and computing $b_{k}=B_{k}/B$ provides the updated value of g(x;s+Δs).

When a lineage is sampled, a new host with one lineage is added to the distribution, and B→B+1. Thus $b_{1}(s+\Delta s)=(1+b_{1}B)/(B+1)$ and for *k* > 1, $b_{k}(s+\Delta s)=b_{k}B/(B+1)$.

### Semi-Parametric Phylodynamic Inference and the Skyspline

In many infectious disease epidemics, incidence of infection through time is likely to change in a nonlinear fashion and potentially very rapidly. We sought to develop a semi-parametric model for the population transmission rate *f*(*t*) which could well describe a large range of epidemic scenarios ranging from exponential growth, SIR dynamics, or endemic equilibrium. We use cubic *akima* splines (Akima 1970) which are robust to large variation in spline coordinates and prevents outlying values. The spline has the following parameters:

- A sequence of time coordinates $\tau_{1}\ldots \tau_{k}$
- A sequence of transmission rate coordinates for log(f(t)): $a_{1}\ldots a_{k}$.

The order of the spline *k* is not determined in advance, but must be estimated. In all experiments, we used a likelihood ratio test to optimize *k*. In order to reduce the number of parameters that must be estimated, we estimate the spline coordinates $a_{1}\ldots a_{k}$, but the spline time coordinates are adapted to the genealogy as follows:

- *τ*_1_ is set to be the TMRCA of the tree
- *τ_k_* is set to be the time of the most recent sample
- The remaining *k* − 2 calibration times are set to correspond to evenly spaced quantiles in the distribution of node heights in the genealogy.

When $f(t;\tau_{1}\ldots \tau_{k},a_{1}\ldots a_{k})$ is specified, the population size can be derived numerically by solving Equation 1. We refer to this model as the *skyspline* model.

A final refinement to the skyspline model is to penalize the likelihood of trajectories if the computed size *y*(*t*) falls below the number of lineages *A*(*t*), which is a logical impossibility for the CoM12 model. For all results presented in this article, likelihoods were heavily penalized: If *A *<* y* and fewer than 20% of coalescent events remain counting from tips to root, the skyspline method will return zero likelihood. The threshold of 20% was chosen so that trajectories with small population sizes subject to stochastic fluctuation would be permitted.

### Coalescent Processes in Large Metapopulations

In Wakeley and Aliacar (2001), the effective population size is derived for a large metapopulation with constant effective size within demes and constant rates of migration between demes and founding unoccupied demes:

(9) $$N_{e}=\frac{y}{2F(\beta +m)}$$

(10) $$F=\frac{1+\beta N/\kappa }{1+\beta N/\kappa +2mN},$$

where *F* is the fixation index which depends on the inoculum size *κ*. The rate of super-infection is denoted *m*, and in our model *m* = 0. In this case F→1 and

$$N_{e}=y/2\beta .$$

This is equivalent to the effective population size as a function of true size and transmission rate derived in Volz (2012) and Dearlove and Wilson (2013). Importantly, this implies that

$$\frac{(\Delta N_{e})/N_{e}}{\Delta t}=\frac{(\Delta y)/y}{\Delta t}$$

so the growth rate of *N_e_* will be the same as *y* even if *N* > 0.

### Simulating Genealogies and the Parametric Bootstrap

Equations 7 and 8 provide a means of simulating genealogies under the multi-scale coalescent process in addition to computing likelihoods. We use Algorithm 1.

**Algorithm 1:** Simulation of genealogy using MSCoM.

**Data**: Sequence of sample times *s_k_*, parameters *θ*

**Result**: Simulated genealogy G

initialization:;

compute *f*(*t*) and y(t)|θ;

start at most recent sample time *s *=* s*_1_ and

initialize G with a single lineage;

**while**G does not have n – 1 internal nodes **do**

Increment time s′=s+Δs.;

Add any lineages sampled in interval

(s,s′) to G;

Compute λ|A,g (Equation 7);

Update *g* in the interval (s,s′) using

Equation 8;

Draw a number of coalescent events

X∼min(Poisson(λ),A(s)−1);

For each coalescent event, randomly

sample two lineages *u* and *v* without

replacement and form new node w=(u,v)

with time s′ to G;

Set s=s′;

**end**

The ability to quickly simulate trees using Algorithm 1 enables a fast approximate parametric bootstrap approach for estimating standard errors and confidence intervals for estimated *y*(*t*) and *f*(*t*) (Volz and Frost 2014). The parametric bootstrap is described in Algorithm 2 and was used for all results. **Data**: Sequence of sample times *s_k_*, estimated parameters $\hat{\theta }$, number of replicates *m***Result**: Estimate variance-covariance matrix of parameters *θ***for**i=1:m**do** Simulate $G^{\left(i\right)}|\hat{\theta }$ using Algorithm 1; Estimate MLE or MAP ${\hat{\theta }}^{\left(i\right)}|G^{\left(i\right)}$; **end** Compute $VCOV({\left\{{\hat{\theta }}^{\left(i\right)}\right\}}_{i=1:m})$; **Algorithm 2:** Parametric bootstrap estimation of variance-covariance of MLE or MAP estimates of parameter vector *θ*.

To generate CI’s for derived quantities such as population size *y*(*t*), we sample *θ* from a multivariate normal distribution centered on the $\hat{\theta }$ with the estimated variance covariance matrix. *y*(*t*) is simulated from each sampled parameter vector and desired quantiles are computed at a given time point.

### Analysis of Latvian HIV Outbreak Data

Data for this analysis were previously described in Balode et al. (2004, 2012) and Graw et al. (2012). These data comprised an alignment of 227 HIV-1 *gag* p17 sequences (HXB2 coordinates 790–1230) collected between 1990 and 2005 from Latvian injection drug users (IDU) and heterosexual sex partners (HET). The Latvian surveillance data were provided by the Infectology Center of Latvia. Previous analyses of the same data (Graw et al. 2012) indicated that the heterosexual and IDU outbreaks were phylogenetically mixed indicating frequent cross-transmission, especially from IDU to HET, so data from both groups was used for phylodynamic analysis. Maximum likelihood phylogenies and 100 bootstrap trees were estimated using PhyML (Guindon et al. 2010) using a GTR+Γ(4)+I substitution model. Each sequence had a known date of sampling so that a molecular clock could be fitted. We used least squares dating (LSD) (To et al. 2015) to fit a molecular clock, root the bootstrap phylogenies, and to rescale bootstrap phylogenies to calendar time.

The skyspline model with either MSCoM or CoM12 likelihoods was used to estimate *y*(*t*) and *R*(*t*). The likelihood was computed as the mean likelihood from a random sample of 20 phylogenies from the PhyML/LSD bootstrap replicates. Estimates were obtained by maximum a posteriori using the simplex optimization algorithm in R. A weak lognormal prior was placed on the death rate (median: 5 years, log standard deviation: 0.75). All other parameters had an improper uniform prior. The parametric bootstrap was used to derive CIs for *y*(*t*) and *R*(*t*) with 120 replicates.

To estimate 2Nμ, estimates of within-host effective size (*N*) were combined with estimates of within-host evolutionary rates on HIV-1 p17 (*μ*) by Zanini et al. (2015). Estimates of *μ* were based on a 500-bp sliding window covering p17 (HXB2 coordinates 760–1260) using serial deep sequencing data from eight patients. To generate credible intervals, we used Monte Carlo integration by repeatedly sampling *N* from the bootstrap distribution and sampling *μ* from a normal distribution using sample means and standard deviations from all eight patients.

### Analysis of EBOV Outbreak Data

Data for this analysis come from a previous phylogenetic analysis by Gire et al. (2014), who estimated time-scaled phylogenies from 78 whole EBOV genomes sampled during the beginning of the 2014 outbreak in West Africa. Phylogenies were estimated by Gire et al. using Bayesian methods (BEAST 1.8) (Drummond et al. 2012), and we use a sample of 40 trees from the posterior distribution for our analysis.

The skyspline model with either MSCoM or CoM12 likelihoods was used to estimate *y*(*t*) and *R*(*t*). The likelihood was computed as the mean likelihood over the sample of 40 posterior trees. Estimates were obtained by maximum a posteriori using the simplex optimization algorithm in R.

A strong lognormal prior was placed on the removal rate (median:15 days, log standard deviation: 0.12), reflecting the large amount of data that have emerged on the natural history of Ebola infection during the West African epidemic (Team 2014). We found that it was difficult to estimate the removal rate with MSCoM and that estimates converged to unrealistically low values. We therefore fixed the removal rate in MSCoM to the MAP estimated gained by CoM12 (rate = 1/10.6 per day). An exponential (rate = 2) prior was used for the within-host effective population size and an exponential (rate = 4) prior was used for the initial number infected. All other parameters had an improper uniform prior. The parametric bootstrap was used to derive CIs for *y*(*t*) and *R*(*t*).

### Simulation Models

In simulation experiments, we consider two stochastic continuous-time epidemiological models, a simple BD process and a more realistic HIV model. In the BD model, $I\overset{\alpha }{\to }2I$ and $I\overset{\beta }{\to }\emptyset$ where *α* = 1 and β=0.5. The within-host population size was assumed to be 2 in units of coalescent time (Sjödin et al. 2005). The HIV model has states *S* for susceptible, *V* for initial infection stage, *A* for acute infection, and *C* for chronic infection. The following reactions govern the system

$$\emptyset \overset{\epsilon }{\to }S$$

$$S\overset{\mu S}{\to }\emptyset$$

$$V\overset{\mu V}{\to }\emptyset$$

$$A\overset{\mu A}{\to }\emptyset$$

$$C\overset{(\mu +\delta_{C})C}{\to }\emptyset$$

$$V\overset{\delta_{A}A}{\to }A$$

$$A\overset{\delta_{C}C}{\to }C$$

$$S\overset{\psi }{\to }A$$

where $\psi =\left(A\beta_{A}+C\beta_{C}\right)\frac{S}{N}\chi$, *ϵ* = 180, $\mu =\frac{1}{30},\;\delta_{T}=365,\;\delta_{A}=1,\;\delta_{C}=\frac{1}{9},\;\beta_{A}=0.5,\;\beta_{C}=0.1,\;\chi =1.5$ giving $R_{0}\approx 2.8$. The within-host populations size is state-specific with parameters $N_{T}=N_{A}=N_{C}=0$ corresponding to no within-host diversity and $N_{T}=0,N_{A}=1,N_{C}=9$ corresponding to high diversity. Note that effective size is reported in units of coalescent time (Sjödin et al. 2005). Sampling in the BD model was concomitant with death, while sampling in the HIV model was homochronous at time 60.

To simulate the viral genealogy we first simulated a transmission history (who infected whom when) from a given transmission model. We then removed all individuals not ancestral to at least one sampled individual. Then, for each individual in a depth-first order, we simulated a within-host genealogy assuming topological neutrality and piece-wise constant population size, propagating any un-coalesced lineages up to the donor.

For the BD process, we can estimate the initial population size, the birth rate, and within-host effective population size assuming death rate is known. In this case, the mathematical method developed in this article is well adapted to this stochastic simulation. However, in the HIV model, we include additional population structure and heterogeneities that are not accounted for in the MSCoM:

- Transmission rate varies over the course of an individual infectious period; five times more infectious in the first year compared with chronic infection.
- Effective population size within hosts also varies over the infectious period. We also include a very shot ‘transmission’ stage that produces a more realistic population bottleneck at transmission.
- We simulate the epidemic in a finite population, and thus the epidemic trajectory is nonlinear. The number of infected hosts initially grows exponentially, saturates, and then slowly decreases.
- There is also natural mortality (one per 30 years per person) and constant birth into the susceptible population (180 individuals per year).

Because of the additional unmodeled complexity in the HIV simulation, we believe this will give a more realistic picture of how the multi-scale coalescent will perform in real-world applications.

## Supplementary Material

Supplementary data are available at *Molecular Biology and Evolution* online.

## Supplementary Material

Supplementary Data
