# Supplementary material for: Phylodynamic Inference across Epidemic Scales
Source: Mol Biol Evol. 2017 Feb 14;34(5):1276–88. doi: 10.1093/molbev/msx077 (PMC5400386; doi:10.1093/molbev/msx077)
Supplement: Supplementary Data [file msx077_Supp.pdf]

Phylodynamic inference across epidemic scales: Supplementary Material

Erik M. Volz, Ethan Romero-Severson, and Thomas Leitner

December 17, 2016

1 Supporting figures

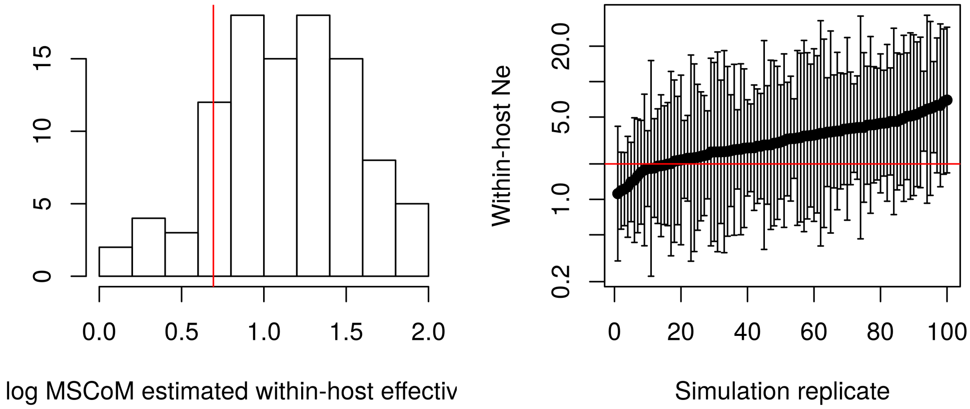

Figure S1: Estimates of within host effective population size using MSCoM applied to exponential growth birth-death model simulated data. Left: Distribution of estimates $\hat{N}$ across all simulation replicates. The true value is indicated by a red line. Right: Estimates of $\hat{N}$ and CIs using parametric bootstrap for all simulation replicates. The true value is indicated by a red line.

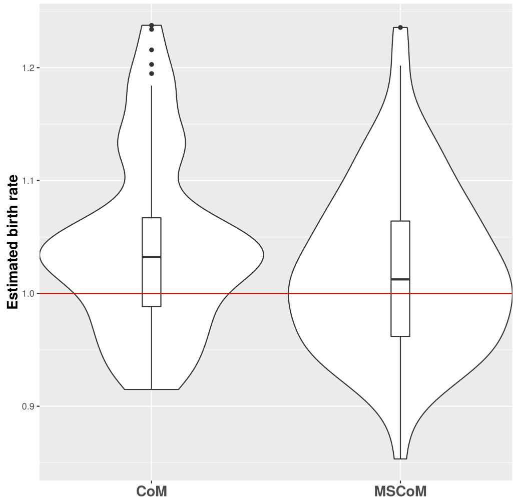

Figure S2: Comparison of CoM and MSCoM estimates of birth rate across all simulation replicates of the exponential growth birth-death model.

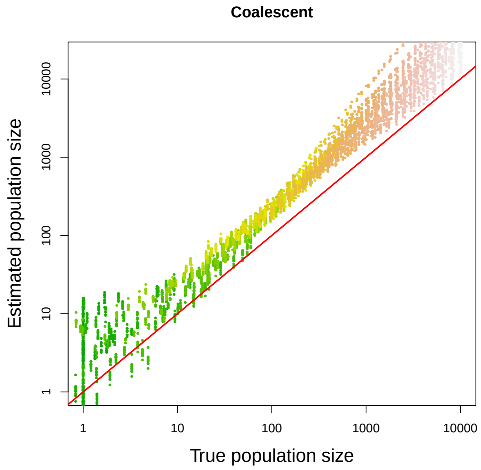

Figure S3: Comparison of the estimated (CoM12) and true population size across all simulation replicates of the exponential growth birth-death model. Colors indicate time in the epidemic when the population size comparison is made. Green corresponds to the early epidemic and red corresponds to the late epidemic.

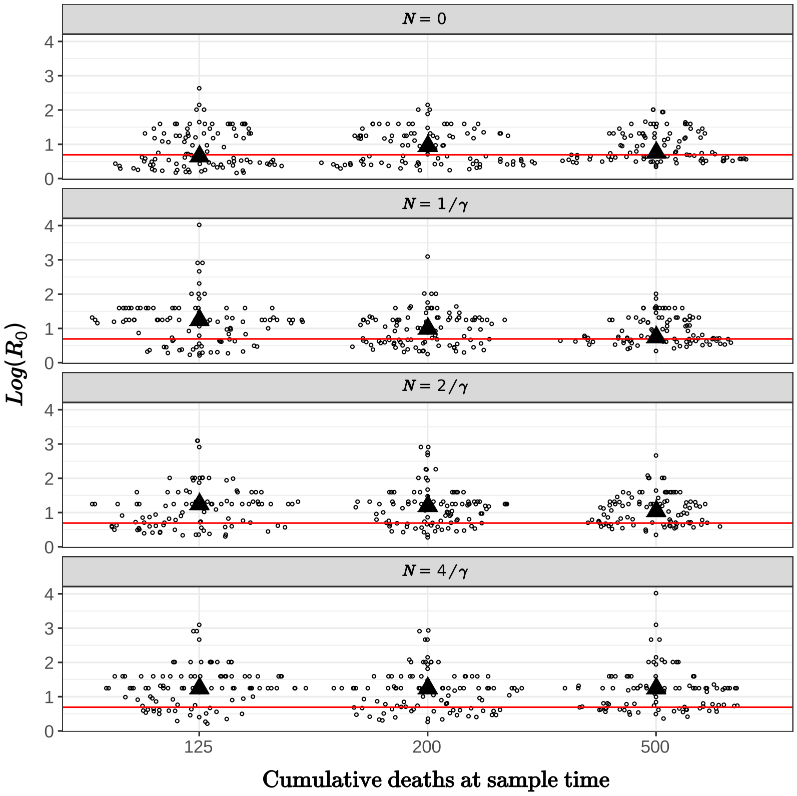

Figure S4: CoM12 estimated reproduction numbers are shown for 100 simulated genealogies from a stochastic birth-death process. Estimates are shown over a range of sample proportions and within-host effective population sizes. The parameter γ denotes the death rate in the birth-death process. The sample size was $n = 100$ in all simulations, and sampling was at a constant rate at time of death. Simulations are grouped along the x-axis according to the number of deaths at the final sampling event. The true reproduction number is indicated by the red line. The triangle indicates the median of all estimates.

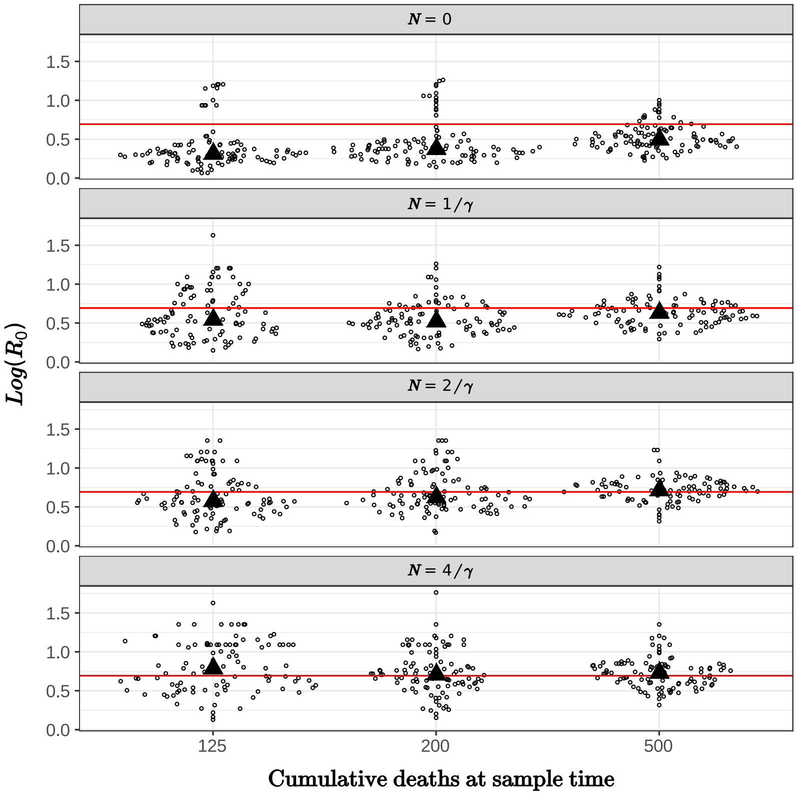

Figure S5: MSCoM estimated reproduction numbers are shown for 100 simulated genealogies from a stochastic birth-death process. Estimates are shown over a range of sample proportions and within-host effective population sizes. The parameter γ denotes the death rate in the birth-death process. The sample size was $n = 100$ in all simulations, and sampling was at a constant rate at time of death. Simulations are grouped along the x-axis according to the number of deaths at the final sampling event. The true reproduction number is indicated by the red line. The triangle indicates the median of all estimates.

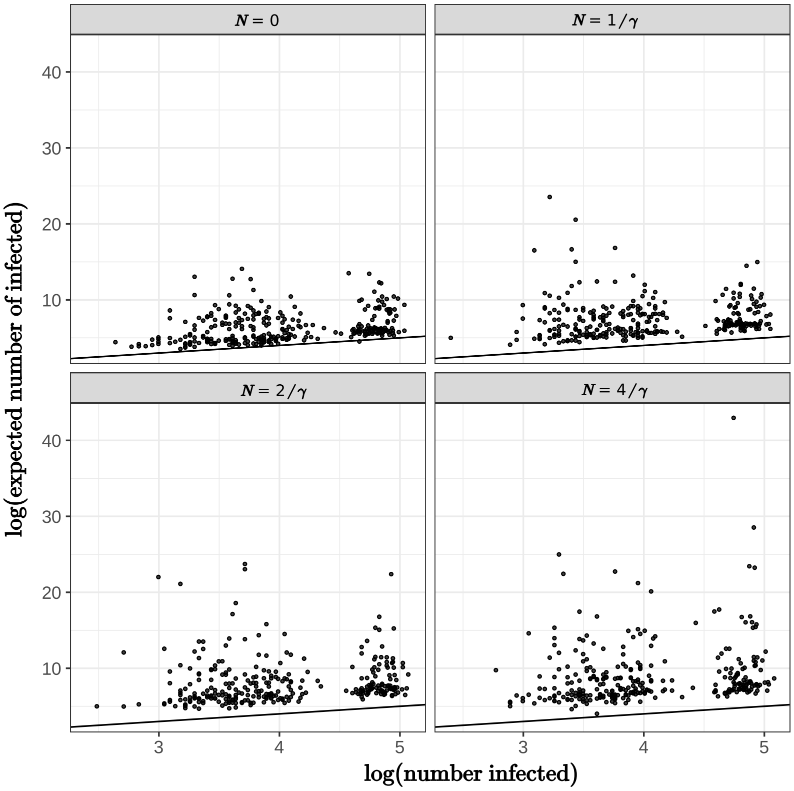

Figure S6: CoM12 estimated number of infected hosts for 100 simulated genealogies from a stochastic birth-death process. Estimates are shown versus the true number of infected hosts from the birth-death simulations. The number infected varies through time and only the estimated number at the time of the last sample is shown. Estimates are shown over a range of sample proportions and within-host effective population sizes. The parameter γ denotes the death rate in the birth-death process. The sample size was $n = 100$ in all simulations, and sampling was at a constant rate at time of death. Simulations are grouped along the x-axis according to the number of deaths at the final sampling event.

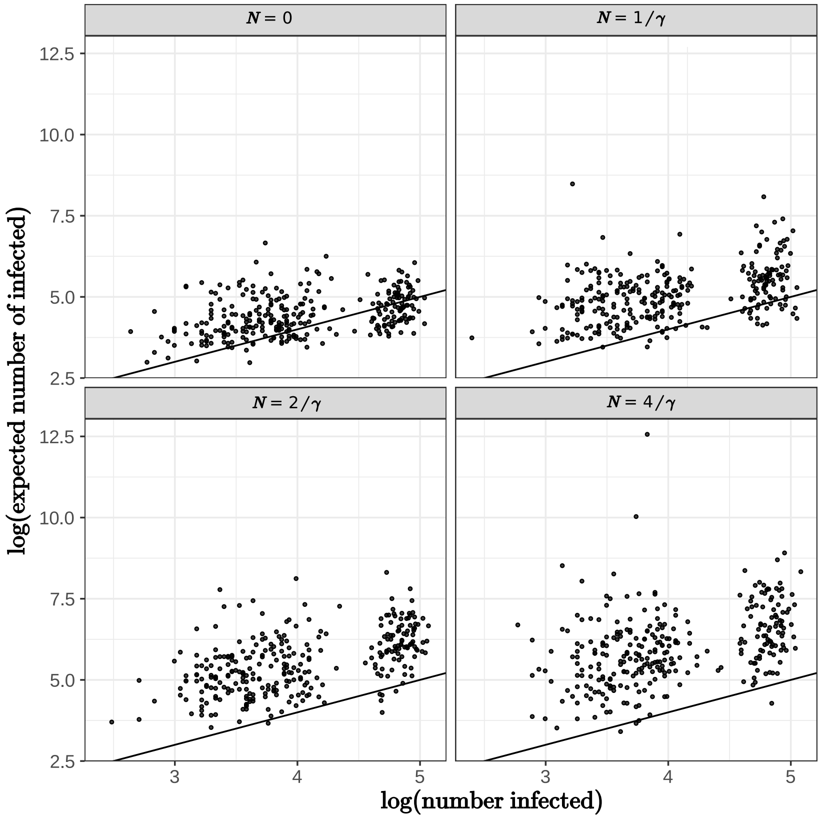

Figure S7: CoM12 estimated number of infected hosts for 100 simulated genealogies from a stochastic birth-death process. Estimates are shown versus the true number of infected hosts from the birth-death simulations. The number infected varies through time and only the estimated number at the time of the last sample is shown. Estimates are shown over a range of sample proportions and within-host effective population sizes. The parameter γ denotes the death rate in the birth-death process. The sample size was $n = 100$ in all simulations, and sampling was at a constant rate at time of death. Simulations are grouped along the x-axis according to the number of deaths at the final sampling event.

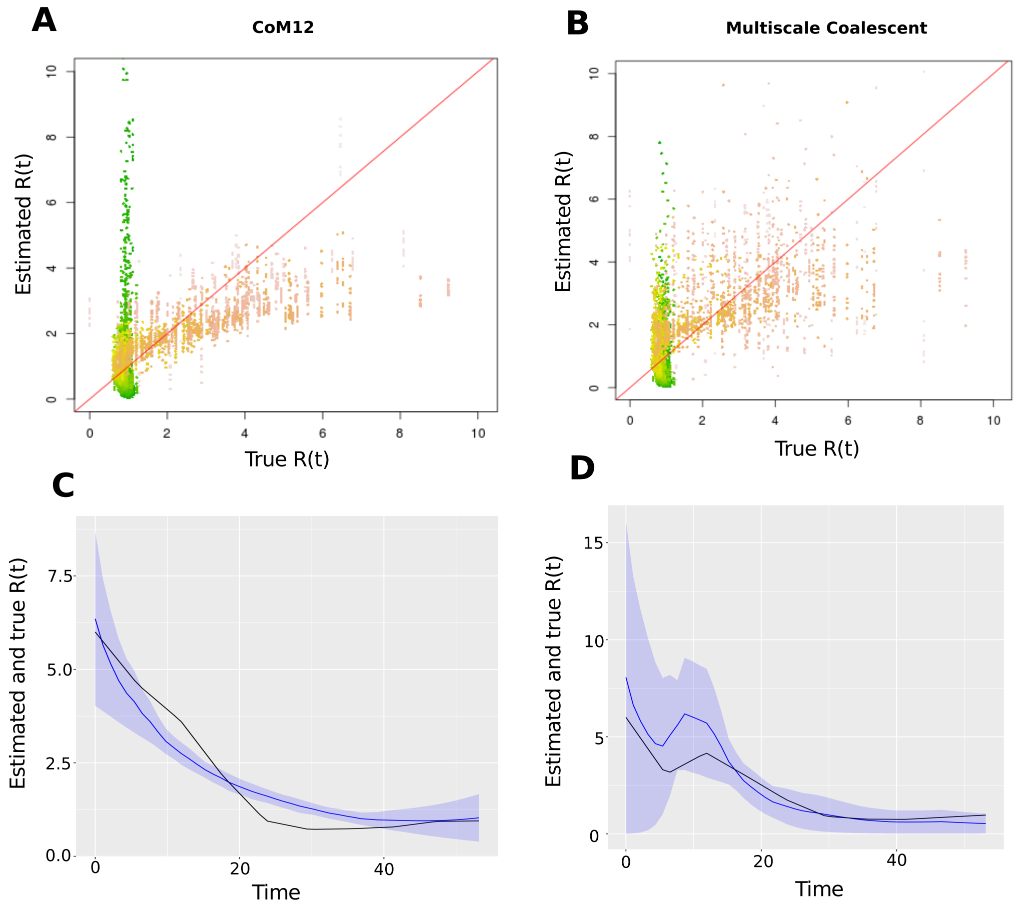

Figure S8: Estimation of reproduction number through time $R(t)$ for HIV simulations. Results are presented for the skyspline/coalescent and skyspline/multiscale coalescent models. A. Comparison of the estimated (skyspline/CoM) and true population size at multiple time points across all simulation replicates. Colors indicate time in the epidemic when the population size comparison is made. Green corresponds to the early epidemic and red corresponds to the late epidemic. B. Comparison of the estimated (skyspline/MSCoM) and true population size at multiple time points across all simulation replicates. Colors are as in (A). C. Example $R(t)$ from HIV simulation (black) with estimates using skyspline/CoM12 (blue) and CI generated by parametric bootstrap approach. D. Example $R(t)$ from HIV simulation (black) with estimates using skyspline/MSCoM (blue) and CI generated by parametric bootstrap approach.

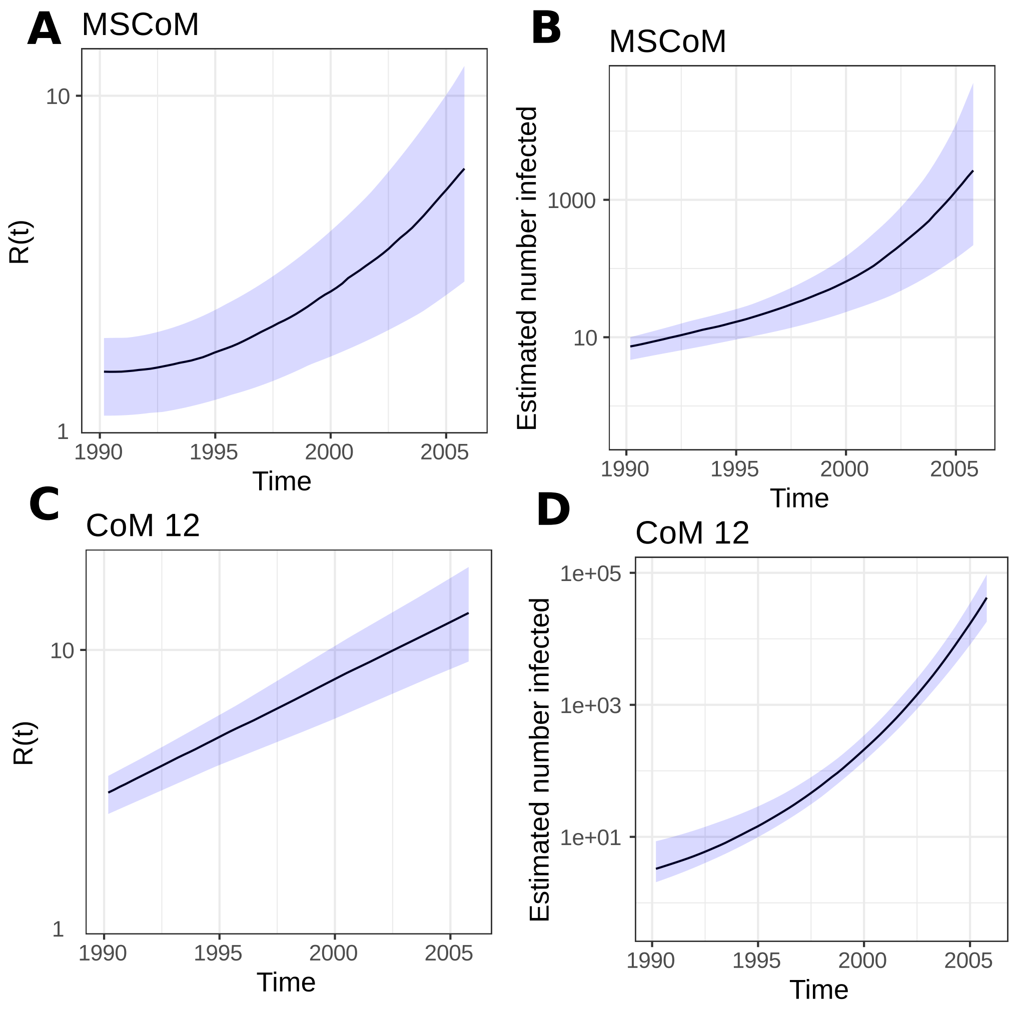

Figure S9: Estimated population size and reproduction numbers for the Latvian HIV data using MSCoM (A & B) and CoM12 (C & D).

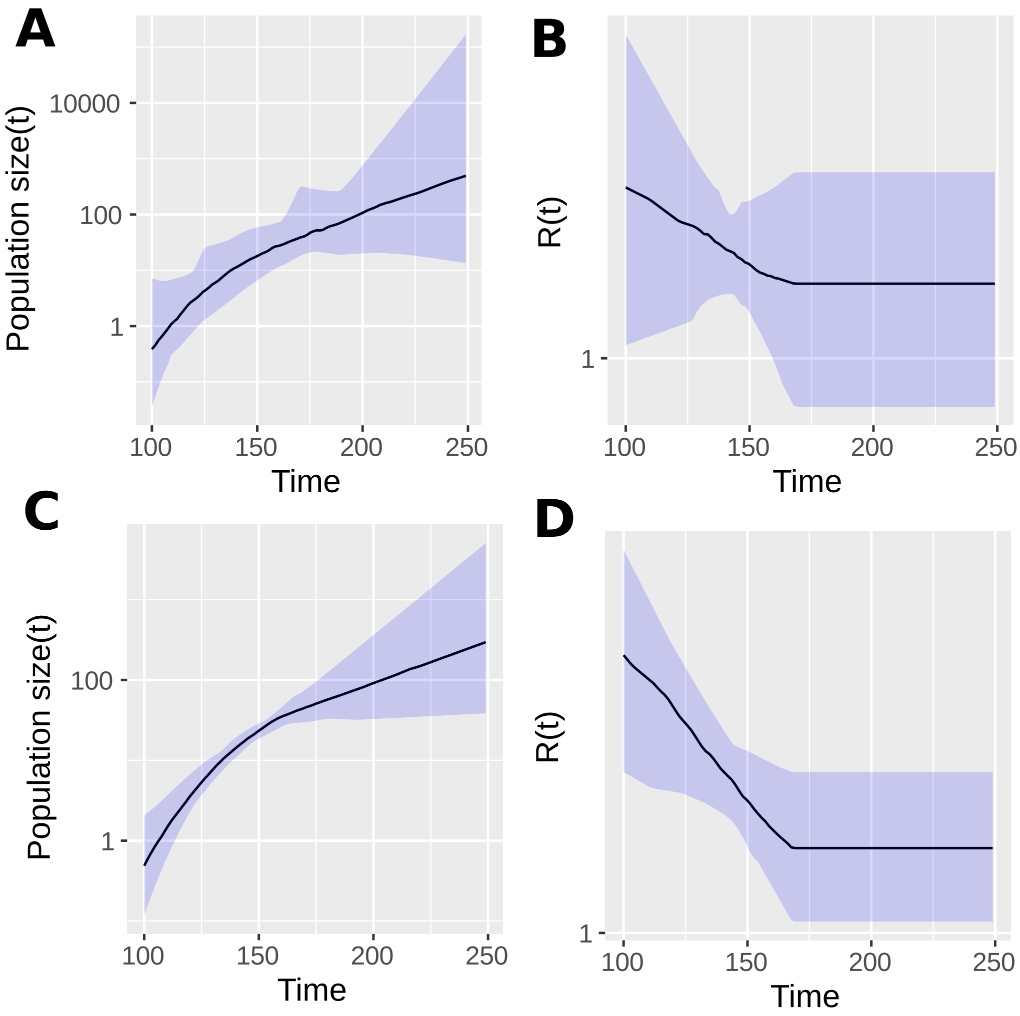

Figure S10: Estimated population size and reproduction numbers for the Ebola data set data using MSCoM (A & B) and CoM12 (C & D). Time is in units of days after 1 January 2014.

2 Alternative derivation of equation 8

In the main text, the following differential equation is derived which describes the time evolution of the distribution of the number of lineages occuping a random infected hosts conditional on being ancestral to the sample:

$$\frac{\delta g(x;s)}{\delta s} = \frac{A(s)f(s)}{g'(1;s)y^2(s)}(g^2(x;s) - g(x;s)) \tag{1}$$

It is also possible to derive the differential equations for the coefficients $b_k(s)$ of $g(x;s)$ without using generating functions. In the following, the time argument (s) will be omited with the understanding that most variables are functions of time. Let the rate of mergers between lineages occupying different hosts be as in the main text

$$\lambda = \binom{B}{2}\frac{2f}{y^2} \tag{2}$$

$$\approx \frac{B^2}{y^2}f \tag{3}$$

where the latter approximation is valid if B is large.

We will define

$$\bar{\Sigma} = \sum_{k'<k} b_{k'} b_{k-k'}$$

which represents the probability that two randomly selected demes will have a total number of lineages $= k$.

Conditional on a merger ocurring, the change in B_k will be as follows

$$\Delta B_k = \begin{cases} +1 & \text{with probabilty } \bar{\Sigma} \\ -1 & \text{with probabilty } 2b_k \\ 0 & \text{with probabilty } 1 - \bar{\Sigma} - 2b_k \end{cases}$$

The dynamics of B_k are

$$\frac{d}{ds}B_k = \lambda \mathrm{E}(\Delta B_k) \tag{4}$$

$$= \frac{f}{y^2}B^2\left(\bar{\Sigma} - 2b_k\right) \tag{5}$$

We now derive $\frac{d}{ds}b_k$:

$$\frac{d}{ds}b_k = \frac{d}{ds}\frac{B_k}{B} \tag{6}$$

$$= \frac{\frac{d}{ds}B_k}{B} - \frac{B_k\left(\frac{d}{ds}B\right)}{B^2} \quad \text{product rule} \tag{7}$$

We use the fact that the number of ancestral hosts decreases at the rate of merges (see main text): $\frac{d}{ds}B = -\lambda$. Then equation 7 becomes

$$\frac{d}{ds}b_k = \frac{f}{y^2}B\left(\bar{\Sigma} - 2b_k\right) + \frac{f}{y^2}Bb_k \tag{8}$$

$$= \frac{f}{y^2}B\left(\sum_{k'<k} b_{k'}b_{k-k'} - b_k\right) \tag{9}$$

which leads to equation 8 in the main text after substituting $B = A/g'$.
